# Genetic Determinants for Metal Tolerance and Antimicrobial Resistance Detected in Bacteria Isolated from Soils of Olive Tree Farms

**DOI:** 10.3390/antibiotics9080476

**Published:** 2020-08-03

**Authors:** Nicolás Glibota, Mª José Grande, Antonio Galvez, Elena Ortega

**Affiliations:** Área de Microbiología, Departamento de Ciencias de la Salud, Facultad de Ciencias Experimentales, Universidad de Jaén, 23071-Jaén, Spain; nag00008@red.ujaen.es (N.G.); mjgrande@ujaen.es (M.J.G.); eortega@ujaen.es (E.O.)

**Keywords:** metal tolerance, antimicrobial resistance, olive farming

## Abstract

Copper-derived compounds are often used in olive tree farms. In a previous study, a collection of bacterial strains isolated from olive tree farms were identified and tested for phenotypic antimicrobial resistance and heavy metal tolerance. The aim of this work was to study the genetic determinants of resistance and to evaluate the co-occurrence of metal tolerance and antibiotic resistance genes. Both metal tolerance and antibiotic resistance genes (including beta-lactamase genes) were detected in the bacterial strains from Cu-treated soils. A high percentage of the strains positive for metal tolerance genes also carried antibiotic resistance genes, especially for genes involved in resistances to beta-lactams and tetracycline. Significant associations were detected between genes involved in copper tolerance and genes coding for beta-lactamases or tetracycline resistance mechanisms. A significant association was also detected between *zntA* (coding for a Zn(II)-translocating P-type ATPase) and *tetC* genes. In conclusion, bacteria from soils of Cu-treated olive farms may carry both metal tolerance and antibiotic resistance genes. The positive associations detected between metal tolerance genes and antibiotic resistance genes suggests co-selection of such genetic traits by exposure to metals.

## 1. Introduction

Antibiotic-resistant (AR) bacteria and their genetic determinants are now being considered as worrisome environmental contaminants, and several studies have shown that natural environments may act as reservoirs of AR bacteria [1,2,3,4]. Not only antibiotics but also other compounds may be responsible for a prolonged pressure that promotes the selection of bacterial antibiotic resistance in different environmental situations, including bioactive compounds such as heavy metals, biocides, detergents, and organic solvents [5,6,7]. Thus, many contaminants could promote antibiotic resistance through co-selection, which could imply co-resistance mechanisms, if different resistance determinants are linked genetically, or cross-resistance, when the same genetic determinant confers resistance to both types of compounds [8]. Among heavy metals, copper has been found to have stronger impacts on antibiotic resistance genes (ARGs) compared to for example iron, lead, nickel and zinc [9,10].

Antibiotic resistance has now become a global issue [11]. The burden of antibiotic resistance may be of greater magnitude when coupled to resistance/tolerance to non-antibiotic compounds. Multidrug-resistant *Salmonella* [12] and methicillin-resistant *Staphylococcus aureus* (MRSA) isolated from animals [13,14] may carry both metal (mainly Cu and Zn) and antibiotic resistance genes. The genetic linkage among different resistance genes suggests that metals could co-select antibiotic resistance in diverse bacterial strains, including human pathogens [15,16].

Previous studies highlight the role of copper contamination in the development of AR bacteria in the environment. Cu-resistant bacteria isolated from soils contaminated with copper were resistance to several types of antimicrobials, including ampicillin, olaquindox, chloramphenicol, tetracycline, and nalidixic acid [17]. Main copper resistance mechanisms rely on export systems based on P-type ATPases or periplasmic detoxification systems such as metallochaperones, multicopper oxidases or resistance-nodulation-division (RND) systems [18]. An increase of both antibiotic resistance and ARGs was reported during copper pollution in source water [19]. Thus, it seems important to reduce not only the use of antibiotics but also the factors that contribute to prevalence of AR bacteria in the environment [20].

Application of copper-derived compounds in olive tree farms is a common practice in South Spain for prevention or treatment of infections caused by the fungus *Spilocaea oleagina*. Phenotypic metal tolerance and AR in bacteria from Cu-contaminated soils in olive tree farms was established in a previous study [21]. The purpose of this study was to evaluate the presence of genetic determinants for metal tolerance or AR in strains isolated from Cu-treated soils in the province of Jaén and classified as resistant to both type of compounds. Possible associations between antimicrobial resistance determinants were also investigated in order to identify gene combinations that may pose a risk for co-selection of resistances.

## 2. Results

### 2.1. Genetic Determinants Coding for Heavy Metal Tolerance and Antibiotic Resistance

The results indicated that 67.7% of the strains carried at least one of the metal tolerance genes investigated (Table 1). Positive results were obtained for the multicopper oxidase gene *copA* (26.04% of strains), the DNA binding repressor protein gene *pcoR* (21.88%), the copper inner membrane pump *pcoD* (10.42%), the outer membrane protein *copB* (2.08%), the periplasmic copper binding protein *copC* (3.13%) and the multicopper oxidase gene *pcoA* (3.13% of strains). The copper export ATPase gene *tcrB* was found in 2.08% of strains, as well as the silver/copper periplasmic metal binding protein gene *silE* (2.08%).

The P-type ATPase *zntA* gene (cadmium, lead and zinc resistances) was found in a high percentage of strains (39.58%). Other metal-tolerance genes detected were *czcD* (13.54%), *merA* (1.04%), *chrB* (8.33%), and *smtA* (9.38%).

Investigation of genes related to antibiotic resistance yielded positive results in 89.58% of the strains (Table 1). 58.3% of the strains presented at least one of the β-lactamase resistance genes studied (*bla*_TEM_, *bla*_PSE_, *bla*_CTX-M_ or *bla*_CTX-M-2_). *bla*_CTX-M_ was the most frequent β-lactamase gene detected (30.21% of the strains), followed by *bla*_TEM_ (15.63%). Genes involved in tetracycline resistance (*tetA*, *B*, *C*, *D*, *E* or G) were also found in 53.1% of strains. The most frequent tet genes were *tetA* (36.46%) and *tetC* (16.67%). Genes coding for export pumps were also detected in resistant strains, although their prevalence was lower. *acrB* gene, involved in the expression of the AcrAB-TolC efflux pump, was the more frequently detected determinant, with 37.5% of positive strains. 7.3% of the strains presented the *aac(6′)-Ie-aph(2″)-Ia* gene encoding the bi-functional aminoglycoside-modifying enzyme. Only 3.1% of strains were positive for *dfrA12* gene, coding for sulfonamide and trimethoprim resistance. The *qacA/B* gene that confers resistance to quaternary ammonium compounds (QACs) and dyes and the deleted *qacE∆1* were detected in 8.3% and 3.1%, respectively. The integrase gene *intl1* associated with class I integrons carrying antimicrobial resistance genes was found in 14.5% of strains.

### 2.2. Association between Heavy Metal Tolerance and Genetic Determinants for Antibiotic Resistance

**Figure 1:**
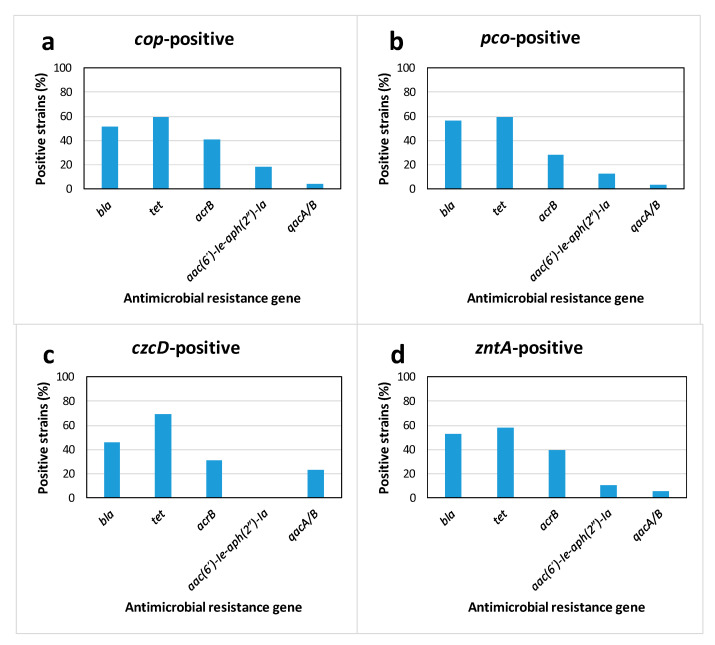
Association between heavy metal tolerance and antibiotic resistance genetic determinants. The percentage of genetic determinants of antimicrobial resistance found in strains that tested positive for heavy metal tolerance genes *cop* (**a**), *pco* (**b**), *czcD* (**c**) or *zntA* (**d**) are shown.

A high percentage of the strains positive for metal tolerance genes also carried antibiotic resistance genes, especially for genes involved in resistances to beta-lactams and tetracycline (Figure 1). Statistically significant associations were detected between *copA* and *tetB/tetC/tetD* (*p* = 0.041) or *bla*_PSE_/*bla*_TEM_ (*p* = 0.01), as well as between *copC* and *bla*_PSE_/*bla*_TEM_ (*p* = 0.008). Significant associations were also found between *pcoR* and *bla*_CTX−M_ (*p* = 0.031). A significant association was also detected between *zntA* and *tetC* genes (*p* = 0.023).

## 3. Discussion

Heavy metals frequently spread on soils by agricultural practices are ubiquitous contaminants highly stable and resistant to degradation. Even low levels of heavy metals have been described as responsible for emergence and dissemination of antimicrobial resistant (AR) strains through co-selection, cross-resistance, co-regulation or biofilm induction [22]. The presence of even low concentrations of antibiotics or toxic compounds in the environment can select for resistance to different antibiotics and heavy metals through a coupled mechanism of resistance against both types of compounds. Reduced cell permeability, target site modification, efflux pump upregulation, acquisition of a neutralizing enzyme with activity against different classes of compounds, as well as physically linked genes responsible for two or more resistances may trigger resistance to more than one antimicrobial agent [23].

The co-selective potential of metals and biocides and its possible role in transmission of antimicrobial resistance genes to human pathogens in different ecosystems needs to be thoroughly investigated [24]. Cu-treated soils from olive tree agricultural fields may represent a source of bacteria subjected to a long-lasting selective pressure by copper and therefore there is a risk for co-selection of antibiotic resistance genes (ARGs).

A possible link between increased Cu levels and the presence of ARGs in agricultural soils subjected to long-term Cu contamination has been previously described [5,10,25]. Cu presence in soils associates positively with certain ARGs even at relatively low levels of the metal [26], suggesting that this metal can increase ARG prevalence even at subtoxic levels.

The present study revealed that the P-type ATPase *zntA* gene was the genetic determinant for metal tolerance more frequently detected among the selected isolates, followed by other metal tolerance genes (e.g., *copA*, *pcoR*, *czcD*, *pcoD*, *smtA* and *chrB*). These results illustrate how soil bacteria from agricultural fields can cumulate different metal-tolerance genes. The results obtained also indicated the presence of ARGs in the metal-tolerant strains. β-lactamase and tetracycline resistance genes were also frequently found in the selected strains.

The analysis of genetic determinants involved in metal tolerances and antibiotic resistances also shows significant associations between *cop* genes and genetic determinants for tetracycline and β-lactam resistances, as well as between *pcoR*, coding for a DNA binding repressor protein involved in copper tolerance, and genes coding for β-lactamases. A significant association was also detected between *zntA*, as well as *czcD*, and genes for tetracycline resistance. Similar relationships between specific metal tolerance and antimicrobial resistance genes were found by Pal et al. [27] from sequence analysis of more than 2500 chromosomes and 4500 plasmids. Both plasmids and genomes with biocide or metal resistance genes more frequently carried antibiotic resistance genes compared to those without biocide or metal resistance genes. Moreover, both types of genes coexisted in bacterial genomes from several environmental sources, including contaminated soils. Copper resistance (*pco*) and β-lactamases (*bla_CTX-M_*) genes coupled in the same plasmid have also been recently described in poultry and pigs in China [28], and copper-tolerant isolates also show the *mcr-1* transferrable colistin resistance gene [29]. These results could suggest that copper may co-select for resistance to antimicrobials considered as an alternative for treatment of multiply resistant strains (such as colistin).

On the other hand, co-selection can also be promoted when different resistance genes are controlled by a single regulatory gene [30]. For example, the expression of the CzcCBA efflux pump and OprD porin (involved in cell permeability to carbapenems) is regulated by CzcR [31].

Previous results further illustrate the promiscuity of the nature of antibiotic resistance among *Bacillus* species and suggest that environmental bacteria may represent a reservoir of resistance genes with the potential to transfer resistance to the food chain or indeed to clinically relevant organisms [32]. Moradali et al. [33] have also summarized several of the well characterized molecular mechanisms which enable *Pseudomonas* species to survive various hostile conditions such as during pathogenesis and antibiotic treatment. These mechanisms form multiple layers of physiological adaptations correlating with social behavior and lifestyle of bacteria while responding environmental stimuli. Such extraordinary adaptive capability relies on extensive numbers of regulatory or controlling factors within integrated and complex signal processing pathways. These enable bacteria to perceive and process environmental cues in order to orchestrate physiological changes to promote adaptation to unfavorable conditions. The ubiquitous presence of *P. aeruginosa* as well as its prevalence and persistence in clinical settings including intrinsic resistance to therapeutics are also attributed to its extraordinary capability of survival by recruiting an arsenal of responsive mechanisms. The genes encoding extended-spectrum β-lactamases and carbapenemase are clinically important not only due to their hydrolyzing activity on a wide range of β-lactams such as carbapenems and extended-spectrum cephalosporins, but also for their worldwide prevalence [34]. The global epidemiology of carbapenem-resistant *P. aeruginosa* was recently analyzed by Hong et al. [35], who reported that the geographical prevalence of extended-spectrum β-lactamases and carbapenemase genes differs from country to country, whereas the genes encoding carbapenemases such as IMP, VIM, and NDM type metallo-β-lactamases have been found in all continents. Recent research [36] points to the environment as an important component for the transmission of resistant bacteria and in the emergence of resistant pathogens. However, a deeper understanding of the evolutionary and ecological processes that lead to clinical appearance of resistance genes is still lacking, which calls for better models of how resistance genes evolve, are mobilized, transferred and disseminated in the environment. Deep analysis of resistance genes in various environmental conditions are of great interest to define the ecological and evolutionary environmental factors that contribute to resistance development and transmission in the community and even in clinical settings.

Resistant isolates of soil origin also efficiently use resistance nodulation-cell division (RND) efflux pumps (EP) [37]. *Stenotrophomonas* spp. environmental strains had been demonstrated to possess similar ARGs as clinical strains [38,39] and similar action of EP in *S. maltophilia* and *Stenotrophomonas* of other species are now described, indicating that the EP present (RND and ABC) are able to confer resistance. Interestingly, it has been found that efflux is also used by *Chryseobacterium* spp. of soil origin, thought these bacteria were mostly know to be resistant by drug modification mechanisms [40].

Hence, resistance mechanisms studies of the most prevalent groups of cultivable bacteria from soils of different farming systems support the significant role of RND and ABC EPs in mediating resistance. The efficient efflux-mediated mechanisms in soil bacteria, therefore, might represent a source for multidrug resistance spread including horizontal transfer [41,42].

Our results may enhance the understanding of the genetics of co-selection and facilitate the evaluation of possible risk scenarios of resistance dissemination in order to reduce the alarming spread of antibiotic resistance genes in the future.

## 4. Materials and Methods

### 4.1. Strain Selection

Heavy metal tolerant bacteria were isolated from soil samples of olive tree farms in a previous study [21]. Strain identification and antibiotic resistance phenotype were established [21] and the 96 strains used in the present study were selected according to soil source and metal tolerance [21].

### 4.2. PCR Detection of Antimicrobial Resistance Genes

The primers and PCR conditions for amplification of antimicrobial resistance genes are detailed in Supplementary file 1. Efflux pump genes studied included *acrB* and *mdfA* [43] and *oqxA* [44]. Genes *qacA/B* (encoding resistance to quaternary ammonium compounds, QACs) were determined according to Noguchi et al. [45]. Amplification of *qacE* and *qacE∆1* genes, their association with Class I integrons, and the presence of the integrase *intl1* gene were studied according to Chuanchuen et al. [46]. Sulfonamide and trimethoprim resistance genes *dfrA12* and *dfrA15* [47] and *aac(6′)-Ie-aph(2″)-Ia* gene involved in aminoglycoside resistance [48] were also investigated.

The beta-lactamase *bla*_PSE_ [49], *bla*_TEM_ [47], *bla*_CTX-M_ and *bla*_CTX-M-2_ [50] and tetracycline resistance genes *tet(A)*, *tet(B)*, *tet(C)*, *tet(D)*, *tet(E)* and *tet(G)* [51] were also studied.

### 4.3. Heavy Metals Tolerance Genes

The multicopper oxidase gene from the plasmid-borne operons *pcoABCDRSE* (from *E. coli*) and *copABCDRS* (from *Pseudomonas syringae*) was amplified according to Kamika and Momba [52] and Badar et al. [53]. *pcoD* (a copper inner membrane pump) [54], and *pcoR*, a DNA binding repressor protein gene [55,56] were also determined.

Other copper-resistance genes investigated included the *cueAR* operon (P1-type ATPase, MerR-type regulatory protein) [57] and *tcrB* (copper export ATPase) belonging to the *tcrYAZB* cluster described in *Enterococcus* [54,58]. The genes *silA* (silver inner membrane proton/cation antiporter) and *silE* (silver/copper periplasmic metal binding protein) of the *silCFBAPRSE* cluster [59], *arsB* (arsenite transmembrane pump) [60] as well as other genes related to metal tolerance such as *merA* (mercuric reductase) [61] and *terF* (tellurite resistance protein) were also studied [60].

The zinc-chromate resistance gene *chrB* was searched according to Nies et al. [62] and Chihomvu et al. [55] and the *czcD* gene (regulation of cobalt, cadmium and zinc efflux system) as described by Medardus et al. [12]. Lead resistance fragment *smtAB* genes were detected according to Naik et al. [63], and *pbrA* was studied for lead resistance [64]. The *ncc* operon was amplified as a fragment that spanned the *nccA* [65]. The Zn(II)-translocating P-type ATPase *zntA* gene described in *E. coli* was investigated according to Rensing et al. [66].

### 4.4. Statistics

Odds ratio (OR) and exact 95% confidence intervals were determined in order to evaluate the association between genetic determinants for heavy metal tolerance genetic and antibiotic resistance. An OR ≤ 1 indicated negative association and OR > 1 positive association.

Differences in the prevalence of antibiotic resistant strains, presence of heavy metal tolerance genes and genetic determinants for antibiotic resistance were analyzed (χ^2^ test). Significance of the association between heavy metal tolerance genetic determinants and both phenotypic resistance to antibiotics or presence of antibiotic resistance genes was analysed by Fisher’s test (*p* < 0.05).

## 5. Conclusions

The present study shows that bacteria from soils of Cu-treated olive farms may carry both metal tolerance and antibiotic resistance genes. The positive associations detected between metal tolerance genes and antibiotic resistance genes suggest co-selection of such genetic traits by exposure to metals.

## Figures and Tables

**Table 1:** Genetic determinants of heavy metal tolerant isolates from agricultural soils.

Species (Isolate)	Heavy Metal Tolerance Genetic Determinants	Antibiotic Resistance Genetic Determinants
*Bacillus cereus* (T22Pb3)	-	*acrB*
*Bacillus endophyticus* (T37Ni3)	-	*tetA*, *tetC*
*Bacillus fordii* (T11Ni1)	*chrB*	-
*Bacillus fordii* (T18Ni1)	*pcoR*, *copA*	*qacA/B*
*Bacillus megaterium* (T1Ni2)	*pcoR*, *chrB*, *copA*	*intl1*, *tetA*
*Bacillus psychrosaccharolyticus* (T4Ni3)	*copA*	*intl1*, *tetA*, *tetC*, *bla*_PSE_
*Bacillus* sp. (T17Ni3)	-	*tetB*, *bla*_PSE_, *bla*_TEM_
*Bacillus* sp. (T19Pb3)	-	*bla*_CTX−M_
*Bacillus* sp. (T20Pb2)	*pcoD*	*acrB*, *aac(6′)-Ie-aph(2″)-Ia*, *tetA*, *tetE*
*Bacillus* sp. (T21Zn1)	*pcoA*	*bla*_CTX−M_
*Bacillus* sp. (T28Pb3)	-	*acrB*, *bla*_TEM_
*Bacillus* sp. (T11Pb1)	-	*acrB*, *bla*_CTX−M_
*Bacillus* sp. (T12Pb1)	-	*acrB*
*Bacillus* sp. (T13Ni2)	-	*intl1*, *tetG*, *bla*_CTX−M_
*Bacillus* sp. (T13Pb1)	-	*acrB*, *bla*_CTX−M_
*Bacillus* sp. (T14Pb1)	-	*intl1*
*Bacillus* sp. (T26Ni3)	*smtA*	*acrB*, *qacA/B*, *intl1*, *dfrA12*, *tetG*, *bla*_CTX−M_, *bla*_CTX−M2_
*Bacillus* sp. (T26Pb2)	-	*acrB*
*Bacillus* sp. (T37Ni2)	-	*dfrA12*
*Bacillus* sp. (T8Pb1)	-	*acrB*
*Bacillus* sp. (T9Ni2)	-	*bla*_CTX−M_, *bla*_CTX−M2_
*Bacillus* sp. (T9Pb1)	-	-
*Burkholderia zhejiangensis* (T37Cd2)	*zntA*	*tetA*
*Burkholderia zhejiangensis* (T38Zn3)	*zntA*	*qacA/B*, *bla*_CTX−M_
*Chryseobacterium formosense* (T11Zn1)	-	*tetA*, *tetC*, *bla*_TEM_
*Chryseobacterium gleum* (T14Cd2)	-	*tetC*
*Chryseobacterium gleum* (T4Ni2)	-	*tetA*, *tetC*, *bla*_CTX−M2_
*Chryseobacterium gleum* (T6Pb1)	*pcoR*, *zntA*	*acrB*, *tetC*, *tetG*, *bla*_CTX−M_
*Chryseobacterium gleum* (T6Zn3)	*pcoR*, *copB*	*tetG*, *bla*_CTX−M_, *bla*_CTX−M2_
*Chryseobacterium hispalense* (T3Zn3)	-	*tetA*, *tetC*, *bla*_CTX−M_
*Chryseobacterium oranimense* (E5Zn1)	*zntA*	*acrB*, *tetD*, *bla*_CTX−M_
*Chryseobacterium oranimense* (T18Cd1)	-	*acrB*, *qacA/B*, *tetA*, *bla*_CTX−M2_
*Chryseobacterium oranimense* (T30Zn1)	-	*aac(6′)-Ie-aph(2″)-Ia*, *tetA*, *tetC*
*Chryseobacterium oranimense* (T31Zn1)	*pcoR*, *zntA*	*tetC*
*Chryseobacterium oranimense* (T37Cd3)	*pcoR*	-
*Chryseobacterium oranimense* (T3Cd1)	-	*acrB*, *tetA*, *tetC*, *bla*_CTX−M_
*Chryseobacterium oranimense* (T3Ni2)	*pcoR*	*tetA*, *tetD*, *bla*_CTX−M_
*Chryseobacterium oranimense* (T3Zn2)	-	*tetA*, *tetE*, *bla*_CTX−M_
*Chryseobacterium oranimense* (T4Cd1)	*pcoR*	*acrB*, *bla*_CTX−M_
*Chryseobacterium oranimense* (T5Cd1)	*pcoR*	*tetA*, *tetC*, *bla*_CTX−M_
*Chryseobacterium oranimense* (T5Ni3)	*pcoR*	*tetA*, *tetC*, *bla*_CTX−M_
*Chryseobacterium oranimense* (T6Cd1)	-	*acrB*, *intl1*, *tetA*, *tetC*, *bla*_CTX−M_
*Chryseobacterium piperi* (T8Cd1)	-	*acrB*, *qacA/B*, *tetA*, *tetC*, *bla*_CTX−M_
*Enterobacter (Klebsiella) aerogenes* (E1Pb3)	*copB*, *copC*, *pcoD*, *tcrB*, *merA*, *zntA*	*bla*_TEM_
*Enterococcus faecalis* (T26Zn2)	*pcoR*	*tetA*, *tetB*, *tetD*, *bla*_CTX−M_
*Flavobacterium johnsoniae* (E1Zn1)	-	*acrB*, *qacE∆1*, *tetA*, *tetC*
*Janthinobacterium lividum* (T25Cd3)	-	-
*Pseudomonas entomophila* (E1Cu3)	*copA*, *pcoA*, *czcD*, *smtA*, *zntA*	*acrB*, *intl1*, *tetA*, *bla*_CTX−M_
*Pseudomonas entomophila* (T11Cd1)	-	*tetA*, *bla*_CTX−M2_, *bla*_TEM_
*Pseudomonas entomophila* (T17Pb1)	*copA*, *zntA*	*acrB*, *tetA*, *tetG*, *bla*_TEM_
*Pseudomonas entomophila* (T23Cu2)	*copA*, *zntA*	*acrB*, *tetA*
*Pseudomonas entomophila* (T34Cu2)	*copA*, *zntA*	*acrB*, *intl1*, *aac(62)-Ie-aph(2″)-Ia*
*Pseudomonas entomophila* (T5Cu2)	*copA*, *smtA*	*acrB*, *bla*_CTX−M_
*Pseudomonas entomophila* (T6Cu1)	*czcD*, *smtA*, *zntA*	*acrB*, *tetA*
*Pseudomonas entomophila* (T7Ni1)	*copA*, *pcoD*, *czcD*, *smtA*, *zntA*	-
*Pseudomonas fluorescens* (E5Zn2)	*copA*, *pcoD*, *zntA*	*aac(6′)-Ie-aph(2″)-Ia*, *tetG*, *bla*_CTX−M_
*Pseudomonas fluorescens* (E5Zn3)	*copA*, *pcoD*, *zntA*	*acrB*, *aac(6′)-Ie-aph(2″)-Ia*, *bla*_CTX−M_, *bla*_PSE_
*Pseudomonas fluorescens* (T12Cu1)	*pcoR*, *pcoD*, *zntA*	*bla*_CTX−M_
*Pseudomonas fluorescens* (T15Cd1)	*chrB*, *czcD*, *zntA*	*tetA*
*Pseudomonas fluorescens* (T25Cu3)	*zntA*	*tetB*, *bla*_CTX−M2_
*Pseudomonas fluorescens* (T25Ni3)	*czcD*	*qacA/B*, *tetG*
*Pseudomonas fluorescens* (T31Cd3)	*czcD*, *zntA*	*qacA/B*, *tetD*, *bla*_PSE_, *bla*_TEM_
*Pseudomonas fluorescens* (T32Cd3)	*pcoR*, *copA*, *zntA*	*tetA*
*Pseudomonas fluorescens* (T35Cd3)	*copA*, *czcD*, *zntA*	*tetA*, *bla*_TEM_
*Pseudomonas fluorescens* (T35Cu2)	*copA*, *pcoD*, *smtA*	*acrB*, *tetA*
*Pseudomonas fluorescens* (T3Ni3)	*copA*, *pcoD*	*tetA*, *bla*_TEM_
*Pseudomonas fluorescens* (T5Cd2)	*pcoR*, *chrB*, *czcD*, *zntA*	*tetA*, *bla*_CTX−M_, *bla*_TEM_
*Pseudomonas fluorescens* (T7Cd3)	*pcoR*, *zntA*	*tetA*
*Pseudomonas lutea* (E3Cu2)	*czcD*, *zntA*	*intl1*
*Pseudomonas lutea* (T8Ni2)	-	-
*Pseudomonas putida* (T1Cu3)	*copA*, *copC*, *czcD*, *smtA*	*acrB*, *intl1*, *tetE*, *bla*_CTX−M_, *bla*_CTX−M2_, *bla*_TEM_
*Pseudomonas* sp. (T15Ni1)	*copA*, *zntA*	*dfrA12*, *tetE*
*Pseudomonas* sp. (T16Ni3)	-	*tetA*, *bla*_PSE_
*Pseudomonas* sp. (T15Cu3)	*copA*, *zntA*	*aac(6′)-Ie-aph(2″)-Ia*, *tetE*, *bla*_TEM_
*Pseudomonas* sp. (T18Cu3)	*copA*, *silE*, *zntA*	*acrB*, *intl1*, *tetA*, *bla*_CTX−M2_
*Pseudomonas* sp. (T19Cu2)	*copA*, *silE,*	*acrB*, *intl1*
*Pseudomonas* sp. (T22Cu1)	*pcoR*, *pcoD*, *zntA*	*tetD*, *tetE*, *tetG*, *bla*_CTX−M_
*Pseudomonas* sp. (T24Cu3)	*pcoR*, *zntA*	*acrB*, *intl1*
*Pseudomonas* sp. (T24Ni1)	*pcoR*, *copA*	*acrB*
*Pseudomonas* sp. (T28Cu2)	*chrB*, *copA*, *zntA*	*acrB*, *bla*_TEM_
*Pseudomonas* sp. (T36Cu3)	*chrB*, *copA*, *czcD*, *smtA*, *zntA*	*intl1*
*Pseudomonas* sp. (T40Cd3)	*czcD*, *zntA*	*qacA/B*, *acrB*, *tetB*, *bla*_CTX−M2_
*Pseudomonas* sp. (T7Cu2)	*pcoD*, *zntA*	*acrB*
*Serratia proteamaculans* (T3Cd2)	*zntA*	*acrB*, *tetB*, *tetE*, *tetG*
*Serratia proteamaculans* (T4Pb1)	*pcoR*, *copA*	*aac(6′)-Ie-aph(2″)-Ia*, *tetB*, *tetG*, *bla*_CTX−M_, *bla*_PSE_
*Sphingobacterium paucimobilis* (T30Zn3)	*pcoR*, *copA*, *copC*	*tetC*, *tetG*, *bla*_PSE_
*Stenotrophomonas maltophilia* (T19Zn1)	*zntA*	*bla*_TEM_
*Stenotrophomonas maltophilia* (T40Zn1)	*zntA*	*acrB*, *bla*_TEM_
*Stenotrophomonas rhizophila* (E1Zn3)	*pcoA*	-
*Stenotrophomonas rhizophila* (T31Zn2)	*zntA*	-
*Variovorax paradoxus* (E4Zn1)	*pcoR*	*acrB*, *tetA*
*Variovorax paradoxus* (T18Zn2)	*czcD*, *smtA*, *zntA*	*tetB*
*Variovorax paradoxus* (T23Zn2)	*zntA*	-
*Variovorax paradoxus* (T32Zn2)	*chrB*	*tetE*
*Variovorax paradoxus* (T32Zn3)	*chrB*	-
*Variovorax paradoxus* (T7Zn1)	-	*tetA*

## Supplementary Materials

The following are available online at https://www.mdpi.com/2079-6382/9/8/476/s1, Table S1: Primers and PCR conditions for amplification of antimicrobial resistance genes.
